# A Feasible Option before Cycle Cancellation for Poor Responders; STOP-START Protocol

**DOI:** 10.22074/IJFS.2021.134626

**Published:** 2021-10-16

**Authors:** Cem Somer Atabekoğlu, Yavuz Emre Şükür, Batuhan Özmen, Murat Sönmezer, Bülent Berker, Ruşen Aytaç

**Affiliations:** Department of Obstetrics and Gynaecology, Ankara University School of Medicine, Ankara, Turkey

**Keywords:** Assisted Reproductive Techniques, Folliculogenesis, Ovarian Stimulation, Unresponsive

## Abstract

Despite the advances in controlled ovarian stimulation (COS), management of a subgroup of poor ovarian responder patients may still be challenging. We describe a feasible and simplified protocol, namely the STOP-START protocol, for poor responders defined as Patient-Oriented Strategies Encompassing Individualize D Oocyte Number (POSEI- DON) groups 3 and 4, who are unresponsive to COS with maximum dose gonadotrophins. Data of 11 women un- responsive to COS were reviewed. Mean age of the patients was 36.5 ± 6.0 years. Unresponsiveness was defined as no follicular growth >9 mm and/or estradiol level less than 40 pg/ml after a week of recombinant follicle stimulating hormone (rFSH, 225-300 IU) administration. In that case, COS was stopped and each woman underwent weekly ul- trasound assessment to catch a secondary follicular growth. All women showed at least one follicular growth within five to 20 days. Six women (54.5%) had spontaneous follicular growth and the other five required ovarian stimulation. At least one oocyte was retrieved from each one of seven patients (63.6%). The mean number of oocytes retrieved was 1.6 ± 1.4 and five women (45.5%) had at least one grade A embryo. Among all, two women became pregnant successfully and both gave live births (18.2%). In conclusion, STOP-START protocol may potentially be an effective, feasible, and time-saving management option for POSEIDON group 3/4 poor responders who are unresponsive to standard COS treatment with maximum dose gonadotrophins.

## Introduction

Despite advances in controlled ovarian stimulation (COS) protocols and laboratory technologies, the management of poor responder patients is still challenging. Recently, the Patient-Oriented Strategies Encompassing Individualize D Oocyte Number (POSEIDON) group suggested a detailed classification system to better identify the poor ovarian response patients who would be included in future studies investigating diagnosis and management (1). Several researches have been conducted and different strategies have been developed to improve the rate of success in assisted reproductive technology (ART) cycles of poor responder patients. However, there is still a subgroup of poor responders who require important decision making: POSEIDON groups 3 and 4 patients who are unresponsive to standard COS treatment with maximum dose gonadotrophins. Therefore, we present the cycle characteristics and outcomes of 11 women managed with the STOP-START protocol.

### Case series

Data of poor responder patients who underwent COS and were unresponsive to stimulation at a university-based infertility clinic between July 2017 and July 2018 were reviewed. The study was approved by the Institutional Review Board of Ankara University (no.: E34690; date: 19.06.2019). POSEIDON group 3 and group 4 poor responders who were unresponsive to COS and were managed by STOP-START protocol were selected from the hospital database. POSEIDON group 3 was defined as patients <35 years, antral follicle count <5 or anti-müllerian hormone (AMH) <1.2 ng/mL and group 4 was defined as patients ≥35 years, antral follicle count <5 or AMH <1.2 ng/mL (1). The inclusion criteria were women aged 18-45 years, a starting dose of gonadotrophin stimulation with 225- 300 IU/day, and unresponsiveness to the first COS. The exclusion criteria were body mass index (BMI) over 30 kg/m^2^ , and the presence of any untreated thyroid dysfunction or hyper-prolactinemia. Eleven patients were eligible for analyses and all data regarding COS, STOP-START protocol, and clinical outcomes were extracted from the hospital database. All women gave written consent for data sharing at the beginning of the COS cycle.

COS was performed with administration of rFSH (Gonal-F, Merck-Serono, Turkey) beginning from the cycle day 2 with a starting dose of 225-300 IU/day. Dose adjustment was performed according to individual ovarian response. As there was no follicular growth, gonadotropin-releasing hormone (GnRH) antagonists were not introduced during the initial stimulation period. The cases were defined as unresponsive to COS when there was no follicular growth >9 mm and/or when the estradiol level was less than 40 pg/ml after a week of maximum dose stimulation by rFSH.

The rFSH treatment was then stopped and each patient underwent weekly ultrasound assessment and blood test beginning after one week to catch a secondary follicular growth (STOP period). When a new wave of follicles grew (at least one >10 mm follicle and/or oestradiol >150 pg/ml), a secondary ART cycle began with spontaneous follow-up (START period). The patients underwent ultrasound and estradiol test in three days and were followed up spontaneously in case of natural follicular development. COS was started with 150 IU gonadotrophin if the existing follicle/s did not show development within three days. GnRH antagonist (Cetrotide, Merck-Serono, Turkey) was commenced (0.25 mg/day) when the leading follicle reached 13-14 mm and continued to grow throughout ovarian stimulation. Transvaginal ultrasound guided oocyte retrieval was performed 35-36 hours after final oocyte maturation with recombinant human chorionic gonadotropin (rhCG, Ovitrelle, Merck, Turkey). A frozen-thawed embryo transfer (FET) was planned due to possible asynchronization of endometrium.

The mean age of the patients was 36.5 ± 6.0 years and all patients fulfilled the POSEIDON criteria for group 3 and 4 (Table 1). The mean AMH level was 0.23 ± 0.25 ng/ mL and the mean antral follicle count was 2.4 ± 1.6. All of the patients were unresponsive to a standard initial COS. Hence, the gonadotrophins were stopped and all patients showed at least one follicular growth within at 5 to 20 days after stopping gonadotrophins.

**Table 1:** The demographics of the study population


Patients (n=11)	Mean ± SD	Min-Max

Age (Y)	36.5 ± 6.0	25-45
Body mass index (kg/m^2^)	25.6 ± 4.3	22-36.8
Baseline E2 (pg/ml)	46.9 ± 30.1	20-113
Baseline FSH (IU/ml)	18.5 ± 8.2	9-36
Baseline AMH (ng/ml)	0.23 ± 0.25	0.01-0.68
Antral follicle count	2.4 ± 1.6	1-6
Duration of infertility (Y)	5.0 ± 3.5	1.5-12
Number of previous IVF attempts	1.3 ± 1.0	0-3


E2; Estradiol, FSH; Follicle stimulating hormone, AMH; Anti-müllerian hormone, IVF; *In vitro *fertilization, Min; Minimum, Max; Maximum, and SD; Standard deviation.

Following the STOP period six patients (54.5%) had spontaneous follicle development within three days and the other five required ovarian stimulation. We were able to retrieve at least one oocyte from each one of seven patients (63.6%) (Table 2). The mean number of oocytes retrieved was 1.6 ± 1.4 and five patients (45.5%) had at least one grade A embryo. As a result, two women got pregnant and both gave live birth (18.2%).

**Table 2:** The cycle characteristics of the study population


	Patients (n=11)	Mean ± SD	Min-Max

1-COS	Duration of stimulation (days)	7.9 ± 2.3	5-12
	Total dose of gonadotrophins (IU)	1955 ± 1033	900-3600
	E2 levels on the day of cancellation (pg/ml)	31.6 ± 10.4	17-42
2-STOP	Duration of cessation period (days)	9.3 ± 4.5	5-20
	Number of follicles >9 mm at return	2.4 ± 0.9	1-4
	E2 levels at return (pg/ml)	178.9 ± 100.5	41-390
3-START	Number of patients with spontaneous follow up (%)	6 (54.5)	
	Maximal E2 levels at follow-up (pg/ml)	332.0 ± 91.9	193-445
	Retrieved oocytes (n)	1.6 ± 1.4	0-3
	Number of MII oocytes	1.1 ± 1.1	0-3
	Fertilization rate (%)	64.2 ± 39.0	0-100
	Number of grade A embryos	0.9 ± 0.9	0-2
	Number of patients with at least one grade A embryo (%)	5 (45.5)	
	Ongoing pregnancy, n (%)	2 (18.2)	


1-COS; Controlled ovarian stimulation, at this step a conventional GnRH antagonist protocol is carried out, 2-STOP; All drugs are stopped, and the spontaneous follicular growth is followed up, 3-START; A new follicular legion had started to grow and followed up spontaneously, or a mild ovarian stimulation protocol was performed, E2; Estradiol, MII; Metaphase II, GnRH; Gonadotropin-releasing hormone, Min; Minimum, Max; Maximum, and SD; Standard deviation.

The first pregnancy was a spontaneous one following oocyte retrieval. The patient was 35 years old and her AMH level was 0.68 ng/ml. Following STOP-START protocol we retrieved two MII oocytes and both were fertilized. She had one frozen grade A embryo and she was called for endometrial preparation on the second day of her next cycle. However, she returned 20 days later with menstrual delay and her βhCG test was positive. 

The second pregnancy was achieved following a fresh embryo transfer in a 29-year-old woman, whose AMH level was 0.09 ng/ml (POSEIDON group 3). Following STOP-START protocol we retrieved three oocytes and only one was MII. Although we opted FET, fresh embryo was transferred by patient demand and in the light of abovementioned patient who got spontaneous pregnancy. This woman received 90 mg/day vaginal micronized progesterone (Crinone 8% gel, Merck-Serono, Turkey) for luteal phase support from the day of oocyte collection until 10 weeks of gestation.

## Discussion

Recently, the wave theory was proposed suggesting that the follicles may be recruited two to three times within a single menstrual cycle, even in the luteal phase (2). Ovarian stimulation in luteal phase results in a longer duration of stimulation and higher total dose of rFSH when compared to conventional start COS (3). The other novel protocols identified by wave theory are randomstart COS and double stimulation (DuoStim) protocols that all support the luteal phase ovarian stimulation (4-7). Hence, in the present series, one of the goals was to reach a new wave of follicular growth. The main difference between STOP-START protocol and DuoStim is the long drug-free interval for approximately one week. However, different from the other protocols evolved from the wave theory, in STOP-START protocol the patient had no ovulation before the spontaneous follicular growth and we actually performed the ovarian stimulation in a prolonged follicular phase. This situation makes fresh embryo transfer possible.

Theoretically, a hormone-receptor complex may be deactivated by external shedding or by internalization of the receptors into the cell. Excess concentrations of trophic hormones, such as FSH, stimulate the process of internalization, leading to a loss of receptors in the cell membrane and thus, a decrease in biological response. During a standard COS, a high dose of rFSH is utilized daily to stimulate the available follicular cohort. Poor responder patients most likely have a reduced number of FSH receptors. In addition, the high-dose rFSH administration in a non-pulsatile manner might reduce the active hormone-receptors by internalization, and at this point the patient becomes unresponsive. The internalized coated pits containing such hormone-receptor complexes are degraded by lysosomes. While the receptors may be reinserted and become functional again, the internalized FSH may mediate biological responses by influencing cellular organelles (8). The suggested mechanism by which the STOP-START protocol possibly works is the prevention of internalization by stopping the rFSH stimulation. Cessation of pushing by FSH (STOP period) probably prevents down-regulation of FSH receptors and allows for the development of some freshened follicles, in which the free receptors are reinserted to the cell membrane and slightly stimulated by the internalized hormone (START period). Protection from a similar down-regulation mechanism of FSH receptors can also be speculated in mild ovarian stimulation cycles and natural cycle *in vitro *fertilization (IVF) procedures for poor responders (9, 10). 

The major strength of the present case series is reporting a feasible stimulation protocol for poor ovarian responder patients. We presented the clinical efficacy of START-STOP protocol with two live births among 11 poor responders. Another strength was presenting the possibility of fresh embryo transfer with this novel protocol. The main limitations of the present case series were the retrospective design and small sample size. The lack of a control group was also noted as a limitation.

## Conclusion

STOP-START protocol might be an effective, feasible, and time-saving management option for POSEIDON group 3/4 poor responders who are unresponsive to COS. However, it’s necessary to confirm the feasibility and effectiveness of this protocol through accomplishing future prospective trials.
